# Occurrence of Ochratoxin A in Southern Spanish Generous Wines under the Denomination of Origin “Jerez-Xérès-Sherry and ‘Manzanilla’ Sanlúcar de Barrameda”

**DOI:** 10.3390/toxins2051054

**Published:** 2010-05-12

**Authors:** Mª Teresa Murillo-Arbizu, Susana Amézqueta, Elena González-Peñas, Adela López de Cerain

**Affiliations:** 1 Department of Nutrition, Food Science, Physiology and Toxicology, School of Pharmacy, University of Navarra, 31008, Pamplona, Spain; Email: mmurarb@alumni.unav.es (M.M.-A.); (A.L.C.); 2 Department of Organic and Pharmaceutical Chemistry, School of Pharmacy, University of Navarra, 31008 Pamplona, Spain; Email: samezqu@alumni.unav.es (S.A.)

**Keywords:** mycotoxins, ochratoxin A, liquid chromatography, wine, Jerez, Xérès, Sherry, manzanilla

## Abstract

The mycotoxin ochratoxin A (OTA) has toxic effects in animals; the most relevant of them is nephrotoxicity. OTA has also been classified as a possible carcinogen for humans (group 2B) by the International Agency for Research on Cancer (IARC). Therefore, exposure to OTA through contaminated food can represent health impairment to humans. The maximum permitted level for this mycotoxin in wine is 2.0 μg/L. The presence of OTA in Spanish wines produced using the traditional methods under the Denomination of Origin “*Jerez-Xérès-Sherry and* *manzanilla Sanlúcar de Barrameda*” was evaluated by a High performance Liquid Chromatography method with fluorescence detection and immunoaffinity column purification. A recovery of 95.4% and a limit of detection and quantification of 0.009 μg/L and 0.02 μg/L respectively, were achieved. In *manzanilla*, *fino*, *amontillado* and *oloroso* wine, the mean OTA values were 0.042, 0.044, 0.144, and 0.319 μg/L, respectively. These levels are not different from other data given in the reference literature on white wines, although *fino* and *manzanilla* wines have very low OTA levels.

## Abbreviations

Body weight (bw); High Performance Liquid Chromatography (LC); Immunoaffinity columns (IAC); International Agency for Research on Cancer (IARC); Limit of Detection (LOD); Limit of Quantification (LOQ); Ochratoxin A (OTA); Phosphate Buffered Saline (PBS); Provisional Tolerable Weekly Intake (PTWI)

## 1. Introduction

Ochratoxin A (OTA) is produced by filamentous fungi of the genera *Aspergillus *and *Penicillium. *The molds that produce OTA in grapes belong to *Aspergillus* section *Nigri*, with *A. Carbonarius* being the most important producer [1]. The growth of mold and the production of this mycotoxin are dependent on several factors such as high temperature and humidity during growth, harvesting, subsequent drying, and food storage. 

OTA is a moderately stable molecule that can remain after most types of food processing and therefore, it appears in a wide variety of foodstuffs. Cereals and cereal products are the main sources of OTA intake, followed by wine, grape juice and coffee [2]. 

The OTA toxic effects have been studied in different animal species, with nephrotoxicity and hepatotoxicity being the most relevant; in addition, and regarding human beings, OTA has been suspected of being involved in the etiology of Balkan Endemic Nephropathy [3], and the International Agency for Research on Cancer (IARC) has classified OTA as a possible carcinogen for humans (group 2B) [4]. Therefore, exposure to OTA through consumption of contaminated food can represent a source of health impairment to humans. Human exposure has been assessed by the analysis of human plasma or serum for OTA, which has revealed the existence of a worldwide chronic exposure to low levels of OTA, including Spain [5]. For a recent review see Pfohl-Leszkowicz and Manderville 2007 [6].

In order to minimize public health risk, European countries have established OTA limits for different food matrices: cereal, coffee, wine and its derivatives. More specifically, the maximum permitted levels for wine (white wine, rosé wine and red wine) is 2.0 μg/L [7], which is in accordance with the proposed maximum OTA levels in wine cited in different studies [8]. 

Wine is a product widely consumed in both developed and developing countries. The presence of OTA in wine was reported for the first time in 1995 [9]. Due to the fact that wine is considered to be a second source (after cereals) of daily OTA intake in human beings [1,10], it is necessary to study the presence of this mycotoxin in wine. Many methods for determining the presence of OTA in this beverage and several studies regarding the presence of this mycotoxin in wine have been carried out worldwide [1,11,12,13,14,15,16,17]. Reference literature has indicated that there is a higher OTA concentration in red wines than in rosé or white wines [11,17]. It should be also pointed out that the further south the origin of the wine samples is in Europe, the higher the frequency of occurrence and the concentration of OTA in red wine [17,18]. 

The OTA levels in wine depend on different factors such as the climate, the date of harvesting and different wine-making procedures [1,5]. A special type of wines are those produced according to the traditional methods as stipulated by the controlling body of the Denomination of Origin “*Jerez-Xérès-Sherry and manzanilla Sanlúcar de* *Barrameda*” in a southern region of Spain, Cadiz. These wines are produced from white grape varieties (mainly *Palomino* and *Pedro Ximenez*) but using different ageing procedures.

During the ageing of *fino* and *manzanilla* wines there is a development of yeast of the *Saccharomyces* genus on the wine surface, which forms a film called, “*blooming veil*” or “*flor veil*”. *Manzanilla* wines are only produced in Sanlúcar de Barrameda (Cadiz, Spain) where the climate conditions allow the yeast to remain alive throughout the entire year. In *fino* wines, the yeast disappears in summer and winter and therefore, the wine alternates biological and oxidative ageing periods. *Amontillado *wines are aged under similar conditions to those of *fino* wines, but when the yeast has disappeared ethanol is added and they are subjected to oxidative ageing. *Oloroso* wines are produced through exclusively oxidative ageing by increasing the ethanol content to 18-20° in the starting wine in order to avoid spontaneous growth of “*flor yeast*”. 

**Figure 1:**
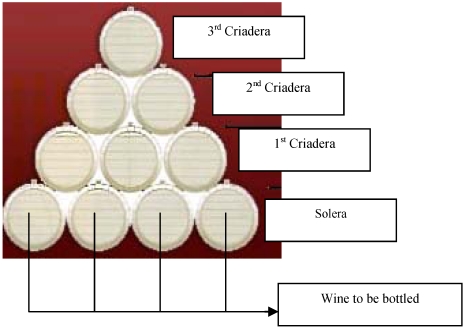
Dynamic System of “Criaderas” and “Soleras” wine ageing.

All these types of wines are aged by following a traditional dynamic system called “Soleras” and “Criaderas” (Figure 1).

These wines are stored in American oak casks piled up in such a way as to form a pyramid, where each level is called *criadera* and the lowest level is called the *solera*. Because yeast requires nutrient renovation in order to survive, there is a periodical mixing of ageing wines with younger wines. The product to be bottled and sold is extracted from the *solera* level casks and after that, the *solera* is replenished by a younger wine from the first *criadera* stage. This process of substitution continues, involving all of the levels. 

The aim of this study was to evaluate the presence of Ochratoxin A in Spanish generous wines (*fino*, *manzanilla*, *amontillado*, and *oloroso*) and also to study the influence that the particular ageing process of these types of wines could have on OTA contamination. The data about OTA contamination in these wines are very interesting because these wines are widely consumed in southern Spain, and large quantities of these wines are also consumed in countries such as Holland, Great Britain, Germany, and the United States, having imported them from Spain.

## 2. Material and Methods

### 2.1. Wine samples

Forty wine samples were analyzed: *fino* (n = 10), *manzanilla* (n = 10), *amontillado* (n = 10) and *oloroso* (n = 10). Due to the particular ageing system, the bottled wine was a mixture of wines from different years. The samples were purchased from supermarkets and small specialized wine shops and analyzed during 2003. The samples were stored in their original bottles at 4-5 °C until analysis was carried out. The day before the analysis, the samples were taken out the fridge in order to temperate them. 

### 2.2. Chemicals and reagents

Ochratoxin A (99.6%) was purchased from Sigma (St. Louis, MD, USA). All of the reagents were pro-analysis grade. Sodium acetate and phosphoric acid were purchased from Panreac (Barcelona, Spain); acetonitrile and methanol LC grade were purchased from Riedel-de Haën (Seelze, Germany). Phosphate buffered saline solution (PBS) was prepared diluting 9.55 g of PBS DULBECCO from Biochrom AG (Berlin, Germany) in one liter of water. Ochratest Immunoaffinity Columns (IAC) were obtained from Vicam Inc. (Watertown MA, USA). Millipore type I water was used to prepare all of the aqueous solutions and was obtained daily from a Milli-Q water purifying system. 

### 2.3. Standard solutions

A stock standard solution of 100 mg/L was prepared by dissolving 1 mg of OTA in 10 mL of methanol. It was then stored at −20 °C. It has been reported that OTA solutions in methanol stored at -20 °C are stable over a period of several years [19]. The exact concentration was determined espectrophotometrically at 333 nm (MW: 403.8; ε: 5500 M^−1 ^cm^−1^) [20]. Working standard solutions and calibration samples were prepared by dilution of stock solution with methanol. Prior to the LC analysis, 200 μL of the calibration samples were evaporated at 40 °C under nitrogen stream and redissolved in 200 μL of mobile phase, in the same way in which the methanol extracts from wine samples were prepared. Quality control of analysis has been carried out by analyzing standard solutions among the wine samples. Moreover, during the years 2002-2005 the laboratory, using the described method, participated in four rounds (1720, 1728, 1736, 1745) for OTA analysis in wine of Food Analysis Performance Assessment Scheme (FAPAS), obtaining satisfactory z scores (|z| ° 2) in each case.

### 2.4. Wine sample preparation and IAC clean-up

The method was based on that which has been described by Otteneder and Majerus (2000) [11], with some modifications. A 5 mL aliquot of wine was diluted with 45 mL of PBS. After shaking, all of the mixture was passed through an IAC column which was preconditioned with 10 mL of PBS. Afterwards, the IAC was washed with 10 mL of PBS and 10 mL of water. Finally, it was dried by passing air with a syringe. OTA was eluted with methanol, 4 times with 1 mL each at a flow rate of 20-30 drops/min. The eluate was evaporated to dryness in a water bath at 40 °C under nitrogen stream, and the residue was redissolved in 200 μL of mobile phase before LC analysis.

### 2.5. Apparatus and chromatographic conditions

The instrument used was an Agilent Technologies 1,100 liquid chromatographic system equipped with a fluorescence detector (model G1321A), controlled by a Chemstation 3D software. The chromatographic conditions were based on the method of Jimenez *et al*. [21]. Briefly, OTA was analyzed on a 5 μm (25 cm × 0.4 cm) Tracer Extrasil ODS2 column with a Tracer Extrasil ODS-2 precolumn, both from Teknokroma (Barcelona, Spain). The injection volume was 100 μL and the flow rate was 1.5 mL/min, with a mobile phase of 29:29:42 (v/v/v) methanol-acetonitrile-sodium acetate (5 mM acidified to pH 2.2 with phosphoric acid). The aqueous phase was filtered through a 0.45 μm membrane filter (Millipore, Ibérica S.A. Spain). Chromatography was performed at 40 °C and the fluorescence conditions were: Ex = 225 nm, Em = 461 nm. in order to achieve a low limit of detection (LOD) [22]. In these chromatographic conditions, OTA has a retention time of 5.5 min. 

## 3. Results and Discussion

### 3.1. Method validation

The selectivity of the method was assured by the use of immunoaffinity purification techniques and a very selective fluorescence detector. Furthermore, some experiments were carried out in order to assess selectivity. When OTA was added to positive samples, an increase of OTA peak area was observed. Moreover, when the proportion of the aqueous component of the mobile phase was increased, the retention time in the OTA peak was delayed, and no other peak, differing from that of OTA, appeared. 

Two calibration curves (seven and eight data points, three replicates for each point) were generated by plotting peak areas of OTA *versus* concentration of calibration samples for two ranges, 0.4 to 1 ng/mL and 1 to 10 ng/mL (equivalent to 0.02-0.04 and 0.04-0.4 ng/mL in wine). Both calibration curves showed a good linear relationship between peak areas and OTA concentration in both ranges. 

Within- and between-day precision and recovery were established by making 27 determinations in spiked wine samples, covering the range of the method (three concentrations of 0.02, 0.1 and 0.4 μg/L/three replicates each one/three different days) and achieving a recovery value of 95.4% ± 5.5%. The limit of detection (LOD) (signal/noise ratio of 3) and of quantification (LOQ) (the lowest concentration assayed that gave good precision and accuracy) were 0.009 and 0.02 μg/L, respectively. Moreover, the good results obtained from FAPAS tests demonstrated the suitability of the analytical methodology for OTA determination in wine. With this method, the laboratory was accredited during the years 2002-2005 for OTA determination in wine by the Spanish Accreditation Board (ENAC) under the Standard ISO 17025.

### 3.2. Sample analysis

**Figure 2:**
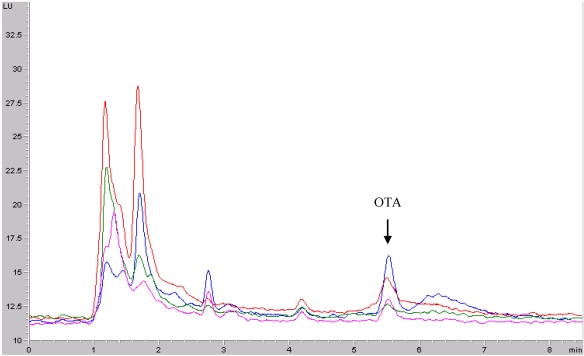
Chromatograms obtained for each type of wine analyzed: *manzanilla *(green), *fino *(pink), *amontillado *(red) and *oloroso *(blue).

Figure 2 shows chromatograms obtained after analysis of samples of each type of wine.

The results obtained from the 40 samples analyzed for OTA content are shown in Table 1.

These results are summarized in Table 2. All of the data has been corrected by recovery (95.4%) and all of them have been taken into account in the calculation, even those values below LOD, in this case, a value of LOD/2 have been assigned.

All of the samples presented OTA concentration levels below the maximum tolerate level (2 µg/L). Thirty-two (80%) samples contained detectable OTA amounts, and the overall OTA mean concentration was 0.138 μg/L. Taking into account the mean value obtained in this study (0.138 µg/L) and the mean obtained from *oloroso* wine (0.319 µg/L), and assuming that a consumer weighs 60 kg with a 50 mL daily intake of these aperitif wines (consumption of this type of wine is occasional and rarely exceeds 50 mL/day [1]), the estimated weekly intakes of OTA are 0.8 and 1.9 ng/kg body weight (bw), respectively, far from the provisional weekly tolerable intake (PTWI) recommended by the Joint FAO/WHO Expert Committee on Food Additives (JECFA) for OTA (100 ng/kg bw) [2].

**Table 1:** OTA levels (μg/L) in wine samples.

Type of wine	Sample number									
	1	2	3	4	5	6	7	8	9	10
*Manzanilla*	0.053	0.039	0.058	0.086	0.048	0.059	0.067	<LOD	<LOD	<LOD
*Fino*	0.092	0.110	0.087	0.084	0.047	<LOD	<LOD	<LOD	<LOD	<LOD
*Amontillado*	0.413	0.132	0.177	0.161	0.151	0.185	0.048	0.049	0.042	0.085
*Oloroso*	0.527	0.278	0.481	0.378	0.642	0.129	0.121	0.265	0.117	0.256

<LOD: below the limit of detection.

**Table 2:** Mean and median OTA levels (μg/L) in wine samples, % of positive samples and range (μg/L).

Type of wine	Mean value		Median		Positive	Range
	(μg/L)	(μg/L)		(%)		(μg/L)
*Manzanilla*	0.042		0.051		70	<LOD-0.086
*Fino*	0.044		0.026		50	<LOD-0.110
*Amontillado*	0.144		0.142		100	0.042-0.413
*Oloroso*	0.319		0.272		100	0.117-0.642
TOTAL	0.138		0.086		80	<LOD-0.642

<LOD: below the limit of detection.

These types of wines are elaborated from white grapes and they are considered to be white wines. The results obtained in this study can be compared with those reported from different studies carried out on white wines in different countries, in which an OTA level range between <LOD-1.72 µg/L was obtained. These are summarized in Table 3. 

Considering all of the wines studied and the fact that they are white wines, they presented OTA levels similar to those obtained in other studies carried out in Spain [5,25], Greece [8,10] or Italy [13]. 

**Table 3:** OTA mean concentration in white wines obtained from different studies.

Origin	% Positive	Mean (μg/L)	LOD (μg/L)	Range (μg/L)	Reference
European and North African countries	27	0.011	0.003	<0.003-0.178	[12]
Worldwide	34	0.07 *	0.01	<0.01-1.20	[14]
	25	0.108	0.01	<0.01-1.36	[11]
	--	0.059	0.05	<0.05-0.18	[23]
	-- ^e^	0.081		<0.05-0.22	
European countries	15	0.012	0.01	<0.01-0.04	[11]
Spain and other European countries	65	0.020	0.003	<0.003-0.267	[24]
Spain	100 ^c^	0.185	0.05	0.154-0.208	[5]
	17 ^d^	0.192		0.192	
	10	0.18	0.05	0.05-1.13	[25]
Italy	28 ª	0.045	0.01	<0.01-0.06	[13]
	100 ^b^	0.535		0.10-0.97	
Morocco	100	0.073	0.01^f^	0.028-0.18	[26]
Greece	55	0.250	0.05	<0.05-1.72	[10]
	54	0.27	0.02	<0.02-0.87	[8]
South Africa	100	0.17	0.01	0.04-0.33	[27]
Turkey	85	0.108	0.006	<0.006-0.618	[28]

^a ^commercial wines; ^b^ homemade wines; ^C ^1997 vintage; ^d ^1998 vintage; ^e ^organically produced; ^f ^limit of quantification; * Median value and not mean value; -- Not indicated.

From all of the wines studied in this work, *manzanilla* and *fino* wines are considered to be aperitif wines. In the reference literature, Burdaspal and Legarda [24] have studied 47 aperitif wines from six different European countries, obtaining an OTA mean concentration of 0.040 μg/L. Among them, Spanish wines (n = 27) presented a slightly higher OTA mean value, 0.054 μg/L. Zimmerli and Dick [12] studied two Sherry aperitif wines (type not mentioned) and obtained 0.042 μg/L as the OTA mean value, similar to the Burdaspal and Legarda data. These reported results coincide with the levels obtained in the *fino* and *manzanilla* wines (OTA mean levels 0.044 μg/L and 0.042 μg/L, respectively).

Comparison of the OTA levels in the four different wine groups was carried out using the median non-parametric test. There are significant differences between the groups *fino*-*oloroso* (p < 0.001) and *manzanilla*-*oloroso* (p < 0.001), while significant differences do not appear between *fino* and *manzanilla* wines (p = 0.656), *fino* and *amontillado* (p = 0.179), *manzanilla* and *amontillado* (p = 0.179) or *amontillado* and *oloroso* wines (p = 0.179).

**Figure 3:**
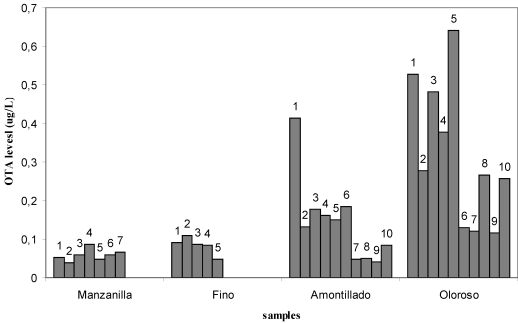
Incidence of OTA in samples.

The wines that suffer biological ageing have OTA levels which are significantly lower than *oloroso* wine, which after suffering an oxidative ageing, have the greatest OTA concentration; and although no significant differences have been observed, it appears that as the biological ageing diminishes, the OTA levels that are obtained tend to be greater. Also, the incidence of OTA in samples is lesser in those wines that suffer a biological ageing (70% in *manzanilla* wines and 50% in *fino* wines) in contrast with the 100% of the samples with detectable OTA levels in *amontillado* and *oloroso* wines, as can be observed in Figure 3. 

Taking into account these considerations, it could be assumed that there is some type of relationship between the presence of OTA in wine and the presence or absence of the blooming veil, because the difference among these wine groups is the ageing process. Different authors have described that yeast can potentially be used for removing OTA from wine [29]. Several hypotheses could be made: yeast could use OTA as a source of carbon for obtaining energy, thereby reducing the OTA levels in these types of wines [17]. Also, yeast or yeast cell walls could adsorb OTA [30], acting like a sponge [18], by different mechanisms such as hydrogen bonding and/or ionic or hydrophobic interactions enhanced by low pH values (near 3.00) [31]; and the wines under study have an average pH of 2.9. Thirdly, it is known that there are certain enzymes with the ability to degrade Ochratoxin A, such as lipase A [32] or carboxypeptidase A [33,34]; these enzymes might be present in yeast, thereby decreasing the OTA level in the medium. 

## 4. Conclusions

All of the samples of wines under denomination of origin “*Jerez-Xérès-sherry and manzanilla Sanlúcar de Barrameda*” analyzed presented OTA concentration levels below the maximum tolerate level (2 µg/L), and in general, the mean OTA value does not appear to differ from other data given in the reference literature regarding white wines and aperitif wines. Considering the low levels of mycotoxin found, the risk for consumers due to OTA exposure through the consumption of this type of wines does not appear to be of great concern. However, it is important to keep in mind that wine is only one of the many possible sources of OTA for humans, and a vigilant attitude must be assumed in order to prevent human intake of OTA from food.

It should be pointed out that there is a tendency to obtain greater OTA levels and incidence of OTA in samples as biological ageing diminishes. Moreover, there are significant differences in the OTA levels between wines with exclusive biological ageing and those with oxidative ageing. This study confirms the importance of taking into account the wine-making procedures when survey studies on OTA in wine are carried out. It has been described in the scientific literature that different yeasts can potentially be used for removing OTA from wine. The special procedure used in the preparation of wines of the Denomination of Origin “*Jerez-Xérès-Sherry and manzanilla Sanlúcar de Barrameda*”, in which naturally occurring yeast appears, seems to be a protection against OTA presence.
